# Effects of sitagliptin on ß-adrenoceptor mediated relaxation in streptozotocin-diabetic rat aorta

**DOI:** 10.3906/sag-2007-234

**Published:** 2021-04-30

**Authors:** Ayhanım Elif MÜDERRİSOĞLU, Betül Rabia ERDOĞAN, Zeynep Elif YEŞİLYURT, Ceren UYAR-BOZTAŞ, İrem KARAÖMERLİOĞLU, Vecdi Melih ALTAN, Ebru ARIOĞLU-İNAN

**Affiliations:** 1 Department of Pharmacology, Faculty of Pharmacy, Ankara University, Ankara Turkey; 2 Department of Pharmacology, Faculty of Pharmacy, Bezmialem Vakıf University, İstanbul Turkey

**Keywords:** ß-adrenoceptors, aorta, streptozotocin induced diabetes, sitagliptin, endothelial nitric oxide synthase

## Abstract

**Background/aim:**

Dipeptidyl peptidase-4 (DPP4) inhibitors, a class of oral antidiabetic drugs, have been shown to be protective on the vascular system because of their antiinflammatory, antiatherosclerotic, and vasodilatory effects. **ß** 2-adrenoceptors **(ß** 2-ARs) mediate the vasorelaxation in the aorta. However, **ß** 3-adrenoceptor-mediated relaxation has not been studied in diabetic aorta yet. Thus, we aimed to study the effect of sitagliptin treatment on **ß** 2- and ß3-adrenoceptor-mediated relaxations in the diabetic rat aorta.

**Materials and methods:**

Eight-week old Sprague Dawley rats were divided into three groups: control, diabetic, sitagliptin treated diabetic. Diabetes was induced by injection of streptozotocin (35 or 40 mg/kg, intraperitoneally). After 10 weeks of diabetes, some of the diabetic rats were treated with sitagliptin (orally, 10mg/kg/day). ß2- and **ß** 3-AR-mediated relaxation responses were evaluated by using isoprenaline and CL 316,243, respectively. **ß3-AR-mediated relaxation** experiments were repeated in presence of L-NAME. Western blotting and immunohistochemistry were performed to determine the abundance of ß3-adrenoceptor and endothelial nitric oxide synthase (eNOS).

**Results:**

The isoprenaline-mediated relaxation response was impaired in the diabetic group and sitagliptin treatment did not improve it. There was no significant change in CL316,243 mediated-relaxation or protein expression of **ß** 3-ARs among the groups. However, the ratio of phosphorylated eNOS/NOS protein was increased markedly in the sitagliptin treated group, which points the stimulating effect of this drug towards the eNOS pathway.

**Conclusion:**

Our results indicate that sitagliptin treatment does not alter **ß-** AR-mediated relaxation in streptozotocin-diabetic rat aorta; however, it significantly stimulates the eNOS pathway. Future studies are needed to clarify the relationship between the eNOS pathway and DPP-4 inhibition.

## 1. Introduction

Chronic diabetes has detrimental effects on the vasculature [1,2]. Antidiabetic drugs with additional vascular protective features have been paid attention in this regard. Dipeptidyl peptidase-4 (DPP4) inhibitors have been shown to exert antiinflammatory, antiatherosclerotic, and vascular protective effects [3,4]. On the other hand, there is an ongoing debate on whether these benefits are independent of metabolic control (euglycemia). Vellecco et al. [5] have demonstrated that linagliptin, a DPP-4 inhibitor, treatment improved relaxation response in nonobese diabetic mice despite the lack of metabolic control. Furthermore, Shah et al. [6] have reported that alogliptin-mediated relaxation was not related to glucagon-like peptide (GLP-1) but rather to nitric oxide (NO) and endothelium-derived hyperpolarizing factor (EDHF) in the aorta of C57BL/6 mice. On the contrary, sitagliptin has been effective in improving the glucose metabolism and endothelial dysfunction in high fat fed-low dose streptozotocin (STZ) injected rat aorta [7]. Hence, this finding was attributed to activation of AMPK signaling pathway through elevated GLP-1 levels. On the other hand, the direct effect of sitagliptin has been tested in the thoracic aorta of New Zealand white rabbits [8]. The drug caused vasorelaxation in the phenylephrine precontracted aorta. This effect has been suggested to be mediated through voltage dependent K+ channels and protein kinase A (PKA). 

Relaxation in thoracic aorta is mediated mainly through ß2-adrenoceptors (ß2-ARs). However, ß3-ARs, the third subtype of adrenoceptors, have been suggested to contribute to vasodilatation through nitric oxide synthase (NOS) and cyclic guanosine monophosphate (cGMP) [9]. Flacco et al. [10] have confirmed this finding as they have shown the presence of ß3-AR and its vasodilator effect in the rat aorta. On the other hand, Brahmadevara et al. [11] have indicated that CL 316,243, a selective ß3-adrenoceptor agonist, failed to induce relaxation in the rat aorta. The reason behind these conflicting results remains unclear.

It is well known that relaxation response in the diabetic aorta is impaired [12–14]. The blunted vasorelaxation response in diabetes has been referred to either endothelium dysfunction [13,15] or decreased ß-AR mediated responsiveness [14,16]. However, the possible alteration in ß3-AR-mediated relaxation due to diabetes has not been investigated yet. 

Thus, the present study has two aims; first, to investigate the effect of sitagliptin on **ß** 2- and **ß** 3-AR-mediated relaxation in STZ diabetic rat aorta and second, to clarify whether this effect is dependent on metabolic control. 

## 2. Materials and methods

### 2.1. Chemicals

Sitagliptin (JANUVIA, 100 mg, Merck Sharp & Dohme, USA); streptozotocin, phenylephrine, isoprenaline, CL 316,243, L-NAME (Sigma, USA); anti- **ß** 3 antibody (ab59685, Abcam, USA), anti-eNOS antibody (9572S, Cell Signaling Technology, USA), anti-phosphorylated eNOS (ser1177) antibody (9571S, Cell Signaling Technology, USA), anti-α-tubulin antibody (ab4074, Abcam, USA), antichicken antibody (29710, AnaSpec, USA), antirabbit antibody (7074, Cell Signaling Technology, USA). 

### 2.2. Animals

The study protocol was approved by the animal welfare committee of Ankara University (permit: 2015-15-172) and was in line with the National Institutes of Health (NIH) guidelines for care and ese of laboratory animals. Eight-week old male Sprague Dawley rats (200–250g) were purchased from Bilkent University and Gazi University, then housed under a 12 h light/night cycle in the animal facility of Pharmacy Faculty of Ankara University. The rats were exposed to food and water Ad Libitum. 

### 2.3. Induction of diabetes and sitagliptin treatment

Rats were divided randomly into three groups; control (C), diabetic (D) and sitagliptin treated diabetic group (S). Diabetes was induced by injection of streptozotocin (35 or 40 mg/kg, intraperitoneally). Their blood glucose was measured with a glucometer after 72 h of injection and rats with blood glucose levels lower than 300 mg/dL received second or, if necessary, the third dose of STZ (40 or 45 mg/kg, respectively). After a 10-week diabetes period, S group was treated with sitagliptin (10mg/kg/day, once a day) for 4 weeks. Sitagliptin doses ranging from 5 mg/kg/day to 50 mg/kg/day have been used in the experimental studies [17–20]. We compared the effectiveness of 10 mg/kg/day [7,19, 21] versus 30 mg/kg/day [7], the most common used doses, on the vasodilatation. Since we did not find a significant difference between the doses, we decided to use the lower dose. For this purpose, Januvia tablets (128.5 mg sitagliptin phosphate monohydrate equivalent to 100 mg sitagliptin) were crushed and suspended in distilled water. The suspension was administered to the rats orally. The C and D groups received distilled water. 

### 2.4. Isolated organ bath experiments

Thoracic aorta was isolated under ether anesthesia and kept in Krebs solution (120 mM NaCl; 4.8 mM KCl; 1.25 mM CaCl2.2H2O; 1.25 mM MgSO4.7H2O; 1.2 mM KH2PO4; 25 mM NaHCO3, and 10 mM glucose monohydrate). Fat and connective tissue was cleaned carefully to avoid damaging the integrity of the endothelium, and the aorta was cut into 4–5 mm long rings. The rings were placed in the organ bath filled with Krebs solution to record isometric force. Two platinum hooks were inserted through the lumen of the aortic rings. One of the hooks was attached to the organ bath chamber. The second hook was attached to an isometric force displacement transducer (MAY, GTA0303, Commat Ltd, Turkey). The transducer was connected to data acquisition system (MAY, MP30B-CE, 310B2428, Commat Ltd., Turkey). The temperature was set at 37 0C and baths were oxygenated continuously (O2 95% CO2 + 5%). The resting tension was set to 2 g. Aorta strips were incubated for 1 h with washing out every 15 min. 

After the incubation period, rings were challenged with 10 µM phenylephrine. Once the contraction response reached to plateau, the integrity of the endothelium was checked with 10 μM acetylcholine. The **ß** 2-AR-mediated relaxation response was determined with isoprenaline (1 nM–10 μM) in the rings precontracted with a submaximal dose of phenylephrine. **ß** 3-AR-mediated relaxation response was evaluated with **ß** 3-AR selective agonist CL 316,243 (0.01 nM–1 μM) and this response was repeated in presence of L-NAME (100 μM).

### 2.5. Western blotting

Thoracic aorta strips were powdered with liquid nitrogen and homogenized with RIPA solution. After sonication, they were agitated for 2 h at 4 0C, and centrifuged at 12,000 rpm for 30 min at 4 0C. Protein concentration was measured using the bicinchoninic acid (BCA) assay. Protein samples (60 μg) were loaded onto polyacrylamide gel (TGX Fast Cast, 7.5%) and transferred to polyvinylidene difluoride (PVDF) membranes. Membranes were blocked with 5% bovine serum albumin (BSA) and incubated with primary antibodies overnight at 4 0C; **ß** 3-adrenoceptor (1/250), eNOS (1/500), phosphorylated eNOS (peNOS) (1/1000, serine-threonine 1177). After washing with tris buffered saline tween 20 (TBST), buffer membranes were incubated with secondary antibodies for 2 h at 4 0C; antichicken, (1/3000), antirabbit, (1/2000). Protein bands were detected with enhanced chemiluminescence assay and exposed to film. Quantification of protein bands was done using Image J (NIH, USA). The expression level was normalized to the housekeeping gene, α-tubulin (1/10000).

### 2.6. Immunohistochemistry 

Aorta tissue samples were fixed in 10% paraformaldehyde solution at least 24 h. Aorta tissues were embedded in paraffin wax, then the tissues were cut into 4–5 µm sections after fixation. To demonstrate the **ß** 3-AR and eNOS protein in the aorta, immunohistochemical analysis was carried out by following instructions of indirect immunoperoxidase streptavidin/biotin kit protocol. Accordingly, specimens were treated with eNOS and **ß** 3-AR antibodies by the streptavidin-biotin immunoperoxidase technique. The color reaction was obtained by using 3-amino-9-ethylcarbazole (AEC) chromogen and Mayer’s hematoxylin counterstain. 

### 2.7. Statistical analysis

The results were expressed as mean ± standard error (SEM). The statistical significance was tested by one way ANOVA. The difference between the groups was tested using the Bonferroni’s multiple comparison test. A value of P < 0.05 was considered significant. 

## 3. Results

### 3.1. General characteristics of the rats

**Figure 1:**
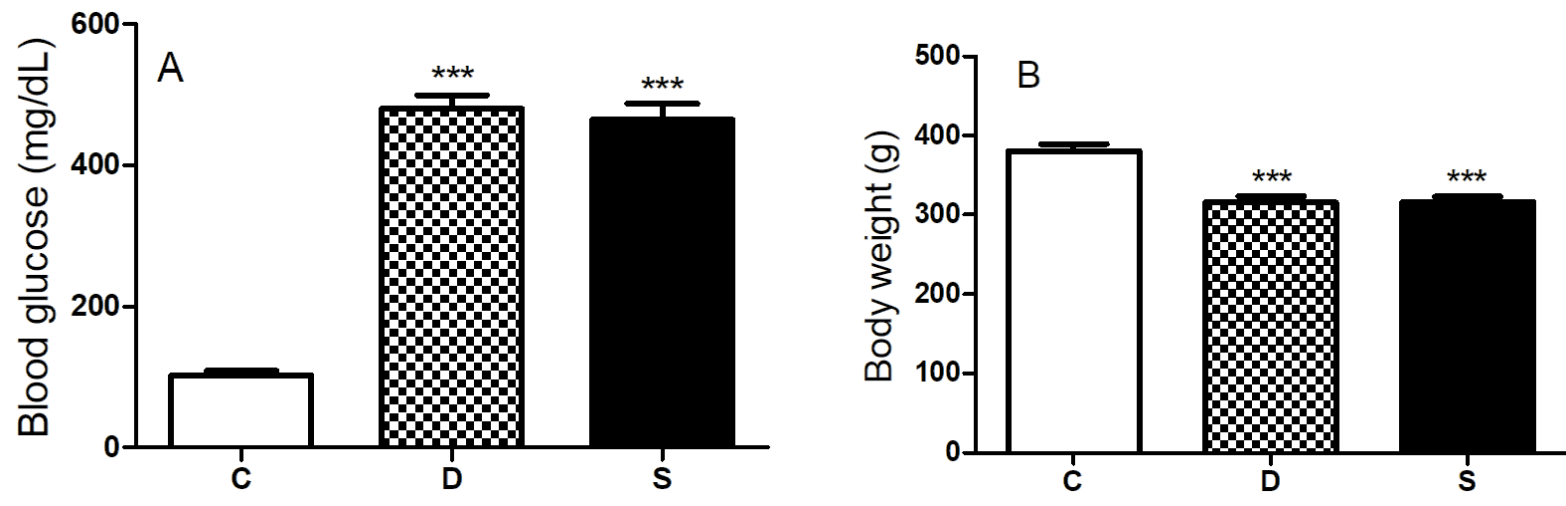
General characteristics of rats. A) blood glucose levels. B) body weights. ***P < 0.001; compared to C group. C, control group; D, diabetic group; S, sitagliptin treated diabetic group.

At the end of the 14-week diabetes period, blood glucose level was higher in diabetic group as expected and sitagliptin treatment did not attenuate it (C: 102.6 ± 5.96 mg/dL; D: 480.1 ± 19.57 mg/dL; S: 465.1 ± 22.49; P < 0.0001) (Figure 1A). In addition, diabetic rats lost weight significantly and sitagliptin treatment did not normalize it (C: 381 ± 8.47 g; D: 315.3 ± 8.55 g; S: 316,4 ± 6.28; P < 0.0001) (Figure 1B). 

### 3.2. ß2- and ß3-AR-mediated relaxation responses in thoracic aorta

**Figure 2:**
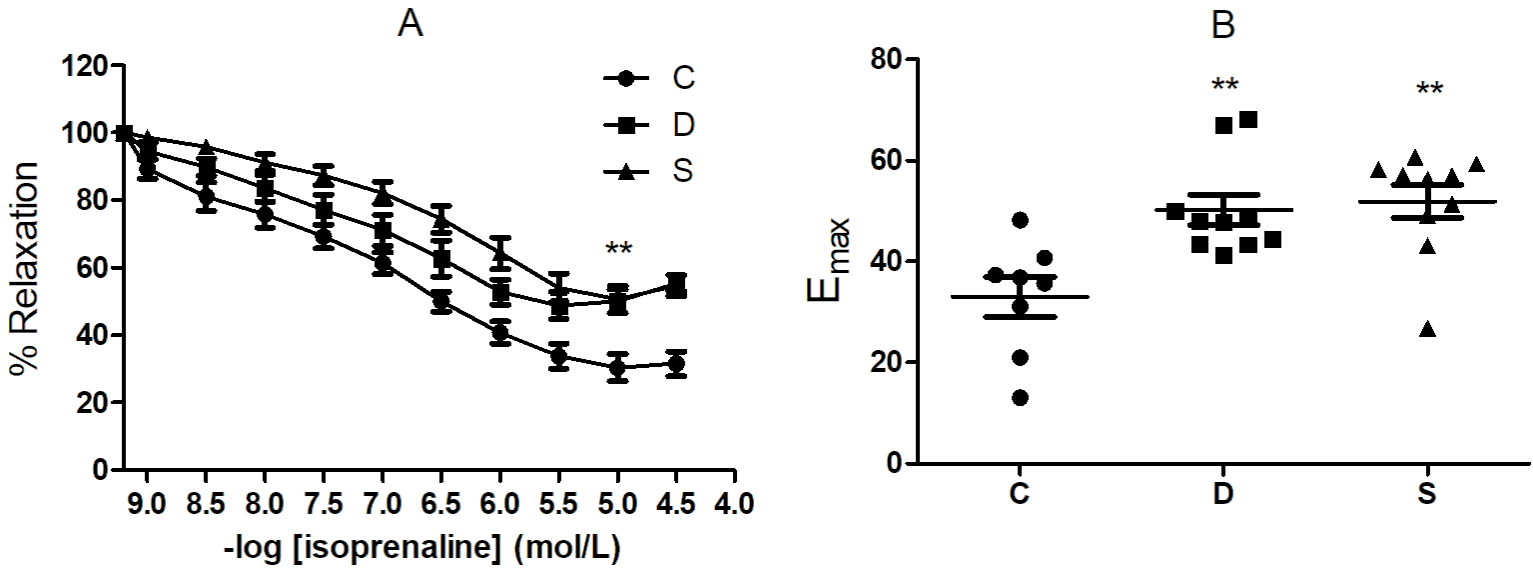
Isoprenaline induced relaxation response. A) cumulative dose response curve. B) maximum response at 10 μM isoprenaline. **P < 0.01; compared to C group. C, control group; D, diabetic group; S, sitagliptin treated diabetic group.

The nonselective **ß** -AR agonist, isoprenaline, induced dose-dependent relaxations in all groups. The responses were markedly lower in the diabetic group and not improved by sitagliptin treatment (Figure 2A). The maximum relaxation response was similar in both diabetic and treated groups (C: 66.73 ± 2.01%; D: 47.99 ± 1.90%; S: 47.62 ± 1.99%; P = 0.0013) (Figure 2B). 

**Figure 3:**
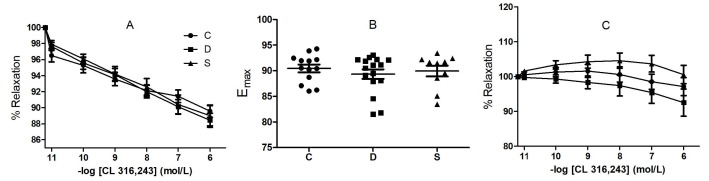
CL 316.243 mediated relaxation response. A) cumulative dose response curve. B) maximum response at 10 μM CL 316,243. C) cumulative dose response curve in the presence of L-NAME. C, control group; D, diabetic group; S, sitagliptin treated diabetic group.

The selective **ß** 3-AR agonist, CL 316,243, induced dose-dependent relaxations in all groups as well (Figure 3A). The maximum response at 10 μM CL 316,243 was not statistically significant among the groups (C: 8.77 ± 0.46%; D: 10.19 ± 0.49%; S: 9.67 ± 0.59%; P = 0.6649) (Figure 3B). The pD2 values, which are the marker for the affinity of the drug to the receptor, of isoprenaline, and CL 316,243 are shown in the Table. The relaxation response of CL 316,243 was abolished in the presence of L-NAME (100 μM, 30 min), an eNOS inhibitor (Figure 3C).

**Table:** The pD2 (logEC50) values of isoprenaline and CL 316,243.

pD2	C	D	S
ISO (%)	7.07 ± 0.10(n = 8)	7.24 ± 0.13**(n = 10)	6.58 ± 0.11**(n = 10)
CL (%)	9.71 ± 0.24(n = 14)	8.98 ± 0.18(n=16)	9.04 ± 0.22(n = 10)

### 3.3. Protein expression levels of ß3-AR, eNOS and peNOS

**Figure 4:**
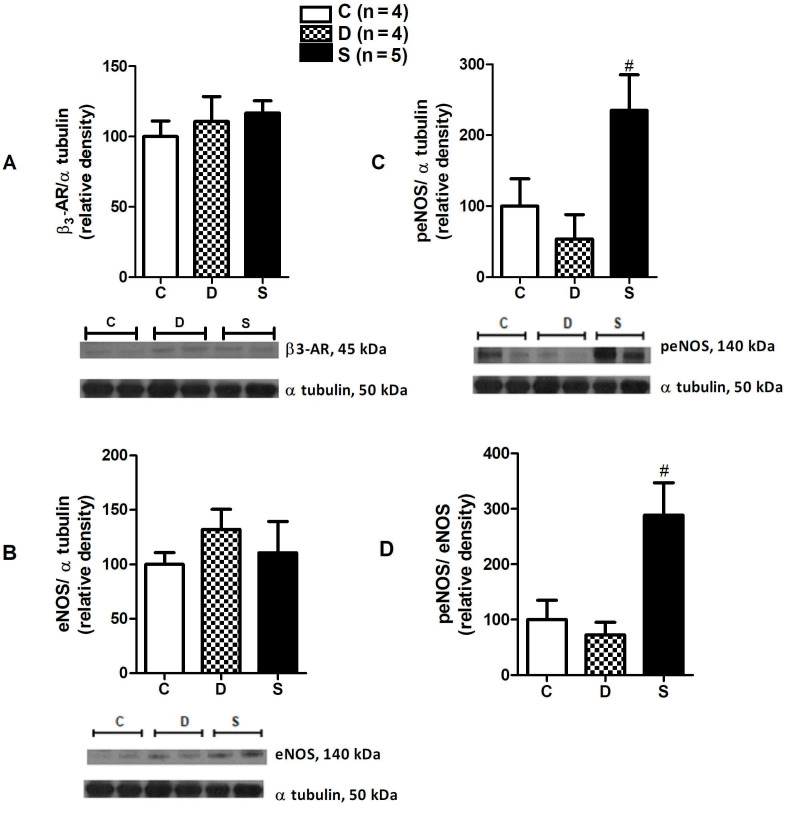
Protein expression levels. A) % relative intensity of ß3-ARs normalized to α-tubulin B) % relative intensity of eNOS normalized to α-tubulin C) % relative intensity of peNOS normalized to α-tubulin D) the ratio of peNOS to eNOS. #P < 0.05; compared to D group. C, control group; D, diabetic group; S, sitagliptin treated diabetic group.

There was no significant change in protein expression of **ß** 3-AR between the groups despite a slight increase in diabetic and sitagliptin-treated diabetic groups (C: 100.00 ± 11.12; D: 110.7 ± 17.9; S: 116.6 ± 8.79; P = 0.6448) (Figure 4A). There was a slight but insignificant increase in eNOS protein expression in the diabetic groups (C: 100.00 ± 10.84; D: 132.0 ± 18.56; S: 110.8 ± 28.69; P = 0.6291) (Figure 4B). On the other hand, eNOS phosphorylation was greater in sitagliptin treated diabetic group (C: 100.00 ± 38.66; D: 53,71±34,49; S: 235.1 ± 50.25; P = 0.028) (Figure 4C). The ratio of phosphorylated eNOS (peNOS) to eNOS was augmented in the sitagliptin treated group (C: 100.00 ± 35.33; D: 72.40 ± 22.91; S: 288.4 ± 58.65; P = 0.0209) (Figure 4D).

### 3.4. Immunohistochemical parameters

**Figure 5:**
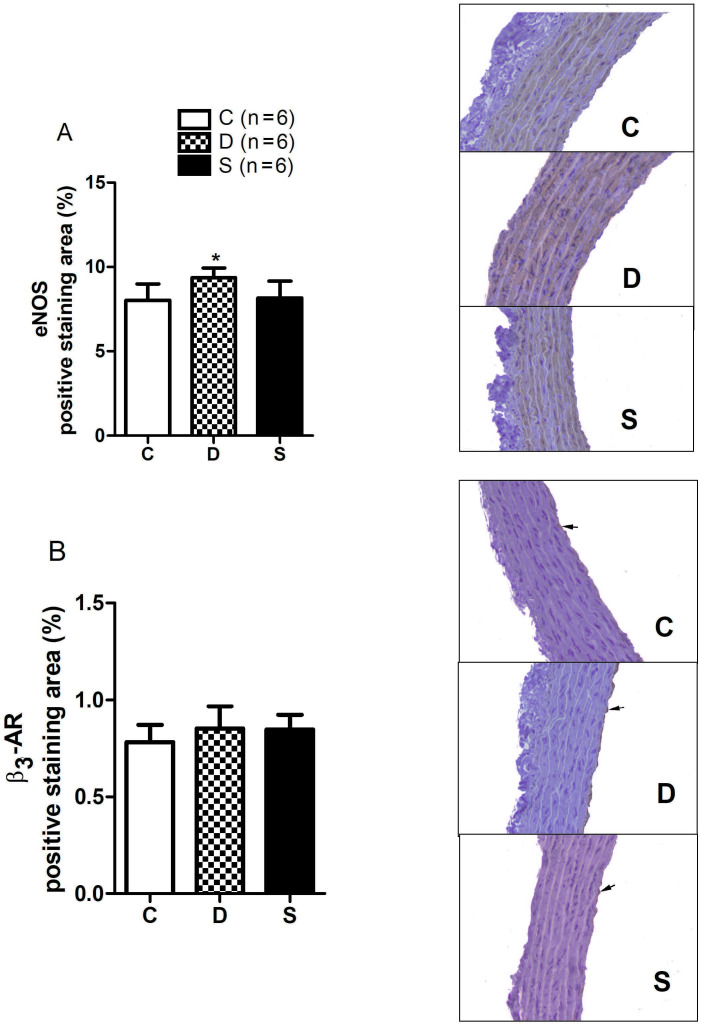
The (AEC) chromogen and Mayer’s hematoxylin counterstain. A) eNOS positive staining area (magnification, 100). B) ß3-AR positive staining area. The arrow indicates the endotelium limited staining (magnification, 100). *P < 0.05; compared to C group. C, control group; D, diabetic group; S, sitagliptin treated diabetic group.

The eNOS immunohistochemical staining observed in the diabetic group was higher compared to the control group. It was reduced in sitagliptin treated group (C: 8.03 ± 0.39%; D: 9.36 ± 0.23%; S: 8.16 ± 0.40%; P = 0.0319) (Figure 5A). The ß3-AR immunohistochemical staining was observed in the endothelial layer and there was no significant difference between the groups (C: 0.78 ± 0.03%; D: 0.85 ± 0.04%; S: 0.84 ± 0.03%; P = 0.3852) (Figure 5B). 

## 4. Discussion

In the present study, we demonstrated that a 4-week sitagliptin treatment did not improve suppressed ß2-adrenoceptor-mediated relaxation response in diabetic rat aorta. Furthermore, we found that ß3-adrenoceptor-mediated relaxation response was not altered in STZ-diabetic or sitagliptin treated STZ-diabetic rat thoracic aorta.

We did not observe a beneficial effect of sitagliptin on isoprenaline-induced vasorelaxation. However, previous studies have reported that DPP-4 inhibitors ameliorated relaxation response in diabetic aorta [5,7,21]. Tang et al. [7] have shown that acetylcholine-induced-relaxation is improved after sitagliptin treatment in high fat fed-low dose STZ injected rats. Furthermore, Wang et al. [21] have confirmed that sitagliptin positively affects acetylcholine-induced relaxation in Zucker Diabetic Fatty (ZDF) rats. However, it should be kept in mind, that both studies have focused on the effects of sitagliptin on endothelium-dependent vasorelaxation. However, different from both studies mentioned above, we investigated the effect of sitagliptin on ß2-AR mediated vasodilatation. The effect of DPP-4 inhibitors on isoprenaline-induced relaxation response was tested with linagliptin by Vellecco et al. [5]. Linagliptin significantly corrected vasorelaxation in type I diabetic Nonobese Diabetic (NOD) mice. In addition, Vellecco et al. [5] have underlined that the effect of linagliptin on vasodilatation is stronger than sitagliptin. 

One possible parameter that affects vasodilator activity of sitagliptin may be the metabolic control. Unlike us, Tang et al. [7] and Wang et al. [21] were able to attenuate blood glucose levels with sitagliptin treatment. However, Vellecco et al. [5] have indicated that they observed improved relaxation response despite the lack of metabolic control. Thus, the beneficial vascular effect of linagliptin has been suggested as independent of glucose control and attributed to its interaction with GLP-1 and GLP-1 receptor. Actually, there is an ongoing debate on whether the positive effects of DPP-4 inhibitors are related to metabolic control or if it is GLP-1 dependent [22]. It is well known that ß2-AR mediated-vasodilatation is blunted in diabetic rat aorta [21]. Similarly, isoprenaline induced-relaxation was impaired in our study and the 4-week sitagliptin treatment did not normalize it. Contrary to our findings, Vellecco et al. [5] have reported the improving effect of linagliptin in NOD mice. Regarding the discrepancy between our findings and those of Vellecco et al. [5], it remains unclear whether it arises from animal species, DPP-4 inhibitor used, or other specific factors in the signaling pathway. It is hard to make further comments as we were unable to investigate the molecular alterations in the ß2-AR-mediated relaxation pathway. Future studies focusing on the effects of sitagliptin on cAMP, PKA or other components in the relavant pathways could help us interpret our findings. 

We also studied ß3-AR mediated vasorelaxation in diabetic rat aorta. We have found that CL 316,243, a selective ß3-AR agonist, caused dose-dependent relaxation in rat aorta. We have also demonstrated that ß3-ARs were localized in the endothelium. Our findings are in line with the previous studies on ß3-AR-mediated-vasorelaxation and the localization of the receptor [9,10,23]. We proposed that the response may be different in diabetic aorta since ß3-AR-mediated cardiac responses have been found to be changed in diabetes [24,25]. Furthermore, the expression of this subtype is upregulated in cardiac pathologies such as diabetes or heart failure [24–29]. However, unlike to the situation in the heart, we found that neither relaxation response nor protein expression of ß3-AR was altered due to diabetes. Moreover, sitagliptin treatment had no impact on these parameters. 

Since ß3-AR-mediated vasodilation has been suggested to be NOS dependent [9,10], we further determined the protein expression and activation of eNOS. The protein expression of eNOS was slightly increased in diabetic and sitagliptin treated group, however, this increase was not statistically significant. On the other hand, the ratio of phosphorylated eNOS to eNOS, a marker of eNOS activation, was significantly increased in sitagliptin treated group. The effect of incretin mimetics on eNOS has been investigated in previous studies. Ding and Zhang [30] have reported that incubation with GLP-1 caused activation and phosphorylation of eNOS in human umbilical vein endothelial cells (HUVEC) after 5 min. Protein expression of eNOS was augmented after 48 h. Thus, our findings demonstrating no significant change in protein expression of eNOS, despite an increased ratio of phosphorylated eNOS to eNOS in sitagliptin-treated rats might be related to the duration of treatment. In addition, both exenatide and GLP-1(9-36) increased the expression, phosphorylation, and activation of eNOS [30]. On the other hand, this effect was partially inhibited when DPP-4 inhibitor sitagliptin or GLP-1 receptor antagonist exendin (9-36) was added. Thus, the effect of GLP-1 on eNOS has been suggested to be related both through GLP-1 receptor and GLP-1(9-36). Supporting this idea, Hu et al. [31] have indicated that the effect of DPP-4 inhibitors on human vascular endothelial cells has both GLP-1-dependent and -independent components. Considering the findings of Ding and Zhang [30], our results on protein expression of eNOS could be attributed to the inhibited conversion of GLP-1 to GLP-1(9-36) by sitagliptin. However, this hypothesis could not explain the augmented eNOS phosphorylation in sitagliptin treated group. On the other hand, our results are parallel to the findings of Matsubara et al. [3]. They demonstrated that eNOS protein expression was preserved, whereas eNOS phosphorylation was markedly increased in des-fluoro-sitagliptin treated apoE deficient high-fat-fed mice. In this study, desfluorositagliptin caused phosphorylation of eNOS via the cAMP/PKA pathway by increasing GLP-1 activity in HUVEC. This phosphorylation further decreased endothelial inflammation, ageing and apoptosis [3]. 

The main findings of our study could be summarized as follows; first, sitagliptin treatment, at least at a dose that does not establish a metabolic control, was ineffective in improving ß2-AR-mediated vasorelaxation in STZ-diabetic rats; second, ß3-AR-mediated relaxation was not changed in diabetic and sitagliptin treated diabetic groups; third, eNOS phosphorylation was augmented in sitagliptin treated group. It remains unclear whether the lack of improved relaxation response by sitagliptin results from the insufficient metabolic control or another cause. Moreover, the significance of the stimulating effect of sitagliptin on the eNOS pathway should be further determined. Future studies with higher doses and longer duration of sitagliptin treatment in comparison to other DPP-4 inhibitors could help to clarify these points.

A limitation of the current study was the lack of a sitagliptin treated control group. A control group with sitagliptin treatment could have been helpful to explain whether the augmented eNOS pathway in sitagliptin treated-diabetic group results from the drug or diabetes itself. However, we were unable to include this group in our study. Another limitation was the lack of a sitagliptin treated diabetic group with metabolic control. In that case, we would have elucidated whether the negative results of the treatment arise from the lack of the metabolic control. Another missing part of the study is the mechanism underlying impaired ß2-AR-mediated relaxation response and augmented eNOS activation. Further studies on the effect of sitagliptin on signaling pathways could help to clarify it.
